# Polyethylene Glycol Loxenatide (PEX-168) Reduces Body Weight and Blood Glucose in Simple Obese Mice

**DOI:** 10.1155/2021/9951463

**Published:** 2021-10-20

**Authors:** Yuting Wu, Zeyuan Guo, Junlu Wang, Yong Wang, Daorong Wang, Ying Li, Lihua Zhu, Xiaofang Sun

**Affiliations:** ^1^Nursing College of Yangzhou University, Yangzhou 225001, China; ^2^Taizhou Hospital of Traditional Chinese Medicine, Taizhou 215300, China; ^3^General Surgery Institute of Yangzhou, Yangzhou University, Yangzhou 225001, China; ^4^Northern Jiangsu People's Hospital, Yangzhou 225001, China; ^5^Clinical Medical College of Yangzhou University, Yangzhou 225001, China

## Abstract

**Background:**

At present, there is a lack of drug treatment for obese patients, so it is needed to find a drug that is effective and has few side effects to treat obesity. PEX-168 is a novel long-acting glucagon-like peptide-1 receptor agonist for T2DM. It improves blood glucose with fewer side effects. The aim of the present study was to investigate the effect of PEX-168 on blood glucose and body weight of mice with simple obesity.

**Methods:**

Thirty healthy and 6-week-old C57BL/6 male mice were randomly divided into a normal control group (NC, *n* = 6) and obesity model group (*n* = 24). The obesity model mice were randomly divided into a high-fat diet group (HF) and intervention groups with different doses of PEX-168 (0.03 mg/kg, 0.1 mg/kg, and 0.3 mg/kg). Each group includes 6 mice. Body weight, food intake, and fasting blood glucose (FBG) were evaluated after intraperitoneal injection, and the intervention was performed weekly for 12 weeks. Fasting insulin (FINS) levels were measured at the 12^th^ week.

**Results:**

Compared with HF, the food intake of mice in the intervention groups decreased transiently, but there was no difference between different doses (*P* > 0.05). The body weight of mice in the middle and high dose of PEX-168 intervention groups decreased significantly, and the differences were statistically significant (*P* < 0.05). The administration of PEX-168 can effectively improve the blood glucose of obese mice, the difference was statistically significant (*P* < 0.05), but there was no difference between different doses (*P* > 0.05). At the 10^th^ week, the incidence of transient hypoglycemia was 67% and 50% in the middle- and high-dose groups, respectively. The levels of serum FINS in the intervention groups were significantly lower than those in the HF group, and the differences were statistically significant (*P* < 0.05), but there was no difference between different doses (*P* > 0.05).

**Conclusions:**

PEX-168 showed significant improvement in the FBG and FINS levels of simple obese mice. Middle and high doses of PEX-168 could reduce the weight of simple obese mice, but there was a certain risk of hypoglycemia.

## 1. Introduction

With the improvement of people's living standards, changed diet structure, and lifestyle, obesity has become a global epidemic [1]. Overweight/obesity increases insulin resistance (IR) and islet *ß*-cell load, leading to increased blood glucose and decreased glucose metabolism, which has a significant impact on the health and quality of life of patients [2]. In recent years, a large number of studies have shown that glucagon-like peptide-1 (GLP-1) and the new-generation hypoglycemic drug glucagon-like peptide-1 receptor agonists (GLP-1 RAs) both have weight loss effect while controlling blood sugar and even have an obvious effect on the treatment of nondiabetic simple obesity [3]. GLP-1 can prevent the function degeneration of islet *ß* cells and increase the sensitivity of islet *a* cells to glucose [4]. GLP-1RAs can increase insulin production by blood glucose levels, improve insulin resistance (IR), regulate blood glucose, and reduce blood lipid and weight at the same time [5].

Natural GLP-1 cannot exist in the human body for a long time, and GLP-1 is degraded by dipeptidyl peptidase 4 (DPP-4) after being secreted and released into blood only 1–2 min [6]. GLP-1 RAs is a GLP-1 analogue which is not easy to be degraded by DPP-4 in contrast. GLP-1 RAs can increase the exogenous GLP-1 concentration, so that the total GLP-1 concentration can reach the pharmacological level without degradation. It has similar biological activity with GLP-1 and is widely used in metabolic diseases [7]. Currently, liraglutide in postmarketing GLP-1 RAs has been approved by the Food and Drug Administration (FDA) as a simple obesity drug due to its excellent weight loss effect and low risk of hypoglycemia in patients with type 2 diabetes mellitus (T2DM) [8].

In GLP-1 RAs, the once-weekly preparation has a longer half-life and stronger stability, which can reduce the number of injections and improve patient compliance. Currently, the weekly injected preparation listed in China mainly includes exenatide microspheres, dulaglutide, and PEX-168. PEX-168 injection was released in the Chinese market in 2019. It is the first self-developed long-term GLP-1 RA in China. PEX-168 can avoid the rapid degradation of DPP-4 enzymes and slow down the metabolism of losenatide and prolong the action time in vivo through the modification of polyethylene glycol, so as to improve the efficacy of the drug [9, 10]. The clinical recommended dose for adults with diabetes starts from 0.1 mg, and the effective dose in rats starts from 0.03 mg/kg. Many studies have confirmed the safety and effectiveness of PEX-168 in the treatment of T2DM patients [11], but the effect of PEX-168 on simple obesity has not been reported. This paper aims to study the effects of continuous administration of PEX-168 for 12 weeks on blood glucose and body weight in mice with nondiabetic simple obesity, so as to provide relevant basis for further research.

## 2. Materials and Methods

### 2.1. Experimental Animals

Animal studies were carried out in twenty-nine C57BL/6 specific-pathogen-free (SPF) male mice, 6 weeks old, and their initial body weights were between 18 and 20 g (Institute of Animal Experiment, College of Veterinary Medicine, Yangzhou University, China; the reference number for the ethical clearance is 2018023). The standard feed and high-fat feed for mice were purchased from the Veterinary College of Yangzhou University and Jiangsu Medison biomedical Co., Ltd. Diet composition is expressed as a percentage of metabolisable energy (ME), control diet: 10% from fat, 20% from protein, and 70% from carbohydrates (ME = 3.85 kcal/g) and high-fat diet: 60% from fat, 20% from protein, and 20% from carbohydrates (ME = 5.24 kcal/g). Animals were housed in a 12-h light/12-h dark cycle and had free access to drinking water and food. The temperature of the animal room was 18–24°C. The humidity was 30%–60%. This experiment has been approved by the Animal Ethics Committee of Yangzhou University.

### 2.2. Main Reagents and Instruments

PEX-168 injections (specification: 0.5 ml, 0.1 mg, provided by Jiangsu Haosen Pharmaceutical Group Co., Ltd., batch No. H20190024) were stored in the dark at 4–8°C. The precision electronic balance was provided by Jiangsu Shuangjie Electronics Co., Ltd., and the blood glucose meter was bought from Roche of Germany.

### 2.3. Measures

#### 2.3.1. Preparation of the Obesity Mice Model

Thirty mice were fed with normal diet for one week. Then, the mice were randomly divided into a normal control group (NC, *n* = 6) and obesity model group (*n* = 24). Six mice were housed in a single cage. The normal control group was fed with normal diet continuously, and the model group was fed with high-fat diet. The calculation formula of obesity degree [11]: obesity degree (%) = (actual weight of the obesity model group−actual weight of the normal diet control group)/actual weight of the normal diet control group *x* 100%. If the obesity degree is greater than 20%, mice in the model group are considered as obese, and the obesity model is established successfully. The weight of mice was measured after 8 weeks of continuous feeding. The obesity degree of all mice in the model group was more than 20%, and the weight did not differ significantly among these mice.

#### 2.3.2. Grouping and Intervention

All 24 obese mice were randomly divided into 4 groups (Table 1): a high-fat diet model control group (HF) and 3 different doses of PEX-168 injection intervention groups (LD group: 0.03 mg/kg; MD group: 0.1 mg/kg; and HD group: 0.3 mg/kg). Each group was given intraperitoneal injection once a week for 12 weeks. During the trial, mice had free access to drinking water and food.

#### 2.3.3. Blood Sampling

After the intervention began, FBG was determined in blood collected from the tip of the tail vein. Twelve weeks later, the mice were fasted for 12 hours, and the blood was collected retro-orbitally. Then, mice were executed using the method of cervical dislocation. The blood was centrifuged at 5000 r/min for 15 min to separate the serum, and the concentrations of FBG and FINS in mice serum were measured with an enzyme-linked immunosorbent assay (ELISA) kit (Jiangsu Synthgene Biotechnology Co., Ltd., Nanjing, China). The remaining serum was stored in −80°C refrigerator for later use. The unit of measurement for the −80°C refrigerator was given by General Surgery Institute of Yangzhou, Yangzhou University.

### 2.4. Statistical Analysis

Statistical analysis was performed using SPSS version 26.0 software. Descriptive data were depicted as means ± standard deviation (SD). Residual analysis was performed to assess normality and homoscedasticity. Data were analyzed using one-way ANOVA followed by Tukey's test. Interrelationships among all indicators were evaluated with the Pearson correlation coefficient (*r*). All results were considered significant at a *p* value < 0.05.

## 3. Results

### 3.1. General Conditions

**Figure 1:**
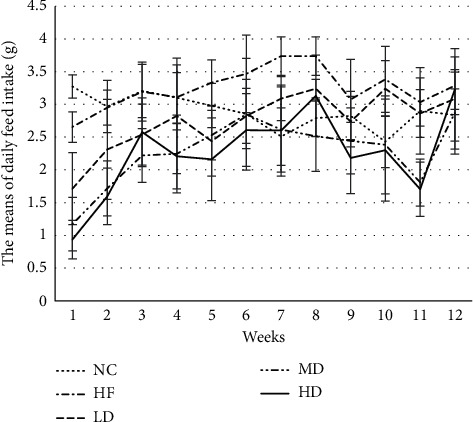
The daily feed intake of each week (means ± SD, g). ^*∗*^Compared with the LD group, the difference was statistically significant (*P* < 0.05).

During the trial, the hair of all mice was black and shiny. The activity and food intake of mice in the PEX-168 injection intervention group decreased within 1-2 days after injection, and the effect gradually decreased as the treatment time extended. As shown in Figure 1, compared with the HF group, the food intake of the intervention group was significantly decreased (*P* < 0.05). Compared with the LD group, the food intake of the MD group and HD groups was significantly different (*P*=0.004 < 0.05 MD; *P*=0.002 < 0.05 HD).

### 3.2. Effects of PEX-168 on Body Weight in Simple Obese Mice

**Figure 2:**
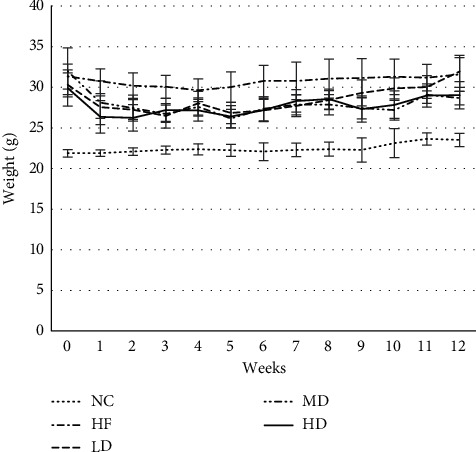
Changes in weight for 12 weeks (means ± SD, g). ^*∗*^Compared with the HF group, the difference was statistically significant (*P* < 0.05).

Before the intervention, the average weight of mice in the NC group was 21.89 g, and the weight of mice in the obesity model group was more than 26.27 g. The obesity degree of all mice was greater than 20%, and the weight did not differ significantly among them. During the intervention period, the weight of mice in the HF group was relatively stable, which met the needs of the follow-up study. After the intervention, the weight of mice in the intervention groups was still higher than that in the NC group, the difference was statistically significant (*P* < 0.05), and the weight loss rate of mice in the three intervention groups was less than 20%. As shown in Figure 2, compared with the HF group, the weight of mice in the MD group and HD group decreased significantly, the difference was statistically significant (*P*=0.009 < 0.05 MD; *P*=0.025 < 0.05 HD), and the weight of mice in the LD group had no significant difference (Table 2).

### 3.3. Effects of PEX-168 on FBG and FINS in Simple Obese Mice

**Figure 3:**
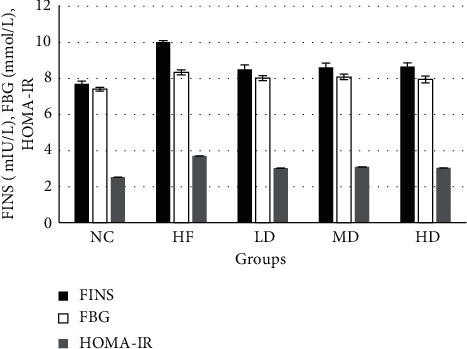
Mean value of FINS, FBG, and HOMA-IR at the 12^th^ week of mice in each group (means ± SD). ^*∗*^Compared with the HF group, the difference was statistically significant (*P* < 0.05).

**Figure 4:**
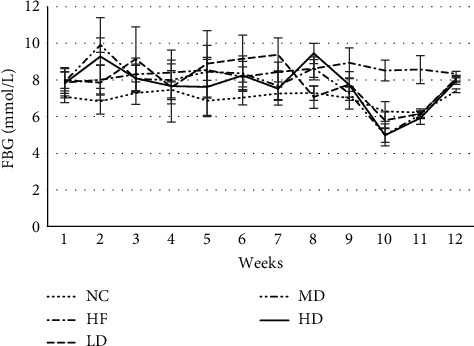
Mean value of FBG measured weekly of mice in each group (means ± SD, mmol/L). The mean value of FBG in the MD group and HD group was lower than 5 mmol/L at the 10^th^ week.

Before the intervention, the mean value of FBG in the NC group was 7.08 mmol/L, and the mean value of FBG in the HF group was 7.89 mmol/L. In this experiment, the normal range of mouse FBG is 7.69 ± 2.60 mmol/L. The abovementioned FBG levels were in the normal range, so the effect of diabetes on this experiment could be excluded. During the trial, the FBG of the HF group did not change significantly, indicating that the simple obesity model was relatively stable, which met the needs of the follow-up study. After 12 weeks of continuous intervention, by computing homeostatic model assessment of insulin resistance (HOMA-IR) [12], the concentrations of FBG, FINS, and HOMA-IR in simple obese mice were significantly higher than those in the NC group, while compared with the HF group, the concentrations of FBG, FINS, and HOMA-IR in the 3 intervention groups were significantly lower (*P* < 0.05). There was no significant difference in the improvement of FBG, FINS, and HOMA-IR among the three different dose groups (Tables 2 and 3 and Figure 3). On the 10^th^ week of the intervention, transient hypoglycemia occurred on mice in the MD group and HD groups with FBG lower than 5 mmol/*L* in 4 and 3 mice, respectively (Figure 4).

## 4. Discussion

Since there are limited drugs to treat obesity patients and GLP-1RAs have the effect on reducing body weight, it is necessary to study the effect of PEX-168 on weight and blood glucose in simple obesity patients, which can provide relevant data reference for weight loss field. A number of studies have confirmed the safety and efficacy of PEX-168 in patients with T2DM [9, 13], while whether PEX-168 can bring a weight loss effect in patients with simple obesity under normal control of blood glucose has not been reported yet.

In this experiment, the subjects of intervention were simple obese mice. Although the biochemical indexes of mice in the obesity model group were higher than those of healthy mice, they were all in the normal range and had not reached the level of diabetes. There was a study reporting that the most common adverse reaction in T2DM patients after using PEX-168 is gastrointestinal reactions [14]. With the prolongation of medication time, gastrointestinal reaction gradually decreased, and gastrointestinal adverse reactions showed obvious dose dependency. Due to that the PEX-168 has the pharmacological effect of slowing down gastric emptying and inhibiting appetite, the food intake of the mice in the intervention groups had a downward trend, and it indicated that the PEX-168 had an influence on the gastrointestinal tract of mice. Although the change is unstable, with the prolongation of the intervention time, the mice gradually tolerate the drug. In this study, compared with the HF group, the MD group and HD group had a significant decrease in food intake and body weight, which indicated that the middle and high dose of PEX-168 had a greater effect on the food intake, so that the weight of the MD group and HD group mice also decreased significantly.

When the glucose level of obese patients reached a certain level, glucose sensitivity is reduced, the insulin concentration in their body also increased, and even hyperinsulinemia and prediabetes symptoms appeared. In this study, after 12 weeks of drug intervention, the FBG, FINS, and HOMA-IR levels of mice in the intervention groups were significantly improved compared with those in the HF group, and the results showed that PEX-168 had a certain regulatory effect on the FBG and FINS levels of simple nondiabetic mice. At the 10th week of the intervention, the MD group and HD group had a transient hypoglycemia event, which returned to the normal level with the intervention. These results indicated that PEX-168 was unstable in regulating blood glucose in simple obese mice. However, the GLP-1RAs hypoglycemic mechanism depends on blood glucose levels in patients regulating islet function [15], so the levels of FBG were returned to normal at the 11th week, and follow-up intervention had a positive effect. In the clinical trials of Shuai et al. [13], T2DM patients' blood glucose levels improved significantly during the treatment of diabetes with PEX-168 and there was no hypoglycemia, which confirmed the safety of PEX-168 regulation of blood glucose in T2DM patients. But, in this study, PEX-168 was used in nondiabetic simple obese mice, and the MD and HD group had hypoglycemia, indicating that, in simple obese mice with normal blood glucose, there is a risk of hypoglycemia, so low doses should be considered as starting quantities. Whether the hypoglycemia events in this experiment were caused by experimental errors needs further experimental study. For example, when blood samples were taken, the mice might have had a stress response.

We acknowledge several limitations to the present study: 1. The experiment did not last long enough. The weights lost of mice were less than 20%, and this may be related to the duration of the intervention. So, the experimental time may need to be extended. 2. This study is a small sample preexperiment. The subsequent experimental research can expand the sample size and reduce the experimental error.

## 5. Conclusions

This study used simple obese mice as model mice and set up different doses of PEX-168 intervention groups and control group to explore the effect of PEX-168 on body weight, blood glucose, and insulin in simple obese mice. The results showed that low-dose PEX-168 could effectively inhibit the feeding of mice, thus improving the FBG of nondiabetic simple obese mice regardless of the dose. PEX-168 can reduce the weight of simple obese mice, but the weights lost of mice were less than 20%. Also, there is a risk of hypoglycemia at moderate and high dose. Thus, follow-up clinical studies may consider setting the concentration of the intervention drug from low doses as initial dosage and from between low- and medium-dose gradients.

## Figures and Tables

**Table 1:** Group of experiments.

Group	Quantity	Intervention	Dosage
NC	6	—	—
HF	6	—	—
LD	6	PEX-168	0.03 mg/kg
MD	6	PEX-168	0.1 mg/kg
HD	6	PEX-168	0.3 mg/kg

**Table 2:** Comparison of body weight, FBG, and FINS in each group (means ± SD). The sample size of NC, LD, and HD groups was reduced from 6 to 5 due to improper operation by the experimenter. The weight and FBG of mice were measured once a week. The levels of FINS were measured at the termination of the study. ^a^Compared with the HF group, the difference was statistically significant (*P* < 0.05); ^b^compared with the NC group, the difference was statistically significant (*P* < 0.05).

Groups	Quantity	Weight (g)	FBG (mmol/L)	FINS (mIU/L)
NC	*n* = 5	23.52 ± 0.90^a^	7.40 ± 0.12^a^	7.70 ± 0.17^a^
HF	*n* = 6	31.57 ± 2.28	8.33 ± 0.15	10.01 ± 0.10
LD	*n* = 5	31.95 ± 2.20^b^	8.02 ± 0.15^ab^	8.50 ± 0.28^ab^
MD	*n* = 6	28.67 ± 0.91^ab^	8.08 ± 0.17^ab^	8.62 ± 0.24^ab^
HD	*n* = 5	29.04 ± 1.87^ab^	7.94 ± 0.21^ab^	8.65 ± 0.25^ab^

**Table 3:** Changes in FBG in each group for 12 weeks (means ± SD, mmol/L). ^a^Compared with the HF group, the difference was statistically significant (*P* < 0.05); ^b^Compared with the NC group, the difference was statistically significant (*P* < 0.05).

Time	NC	HF	LD	MD	HD
1w	7.08 ± 0.38	7.85 ± 0.64	8.00 ± 0.50^b^	7.90 ± 0.84	7.82 ± 0.88
2w	6.84 ± 0.86	8.02 ± 0.85	7.86 ± 1.07	9.90 ± 0.43^ab^	9.28 ± 2.31^b^
3w	7.28 ± 0.75	8.30 ± 0.98	9.14 ± 1.97^b^	8.05 ± 0.81	8.09 ± 0.84
4w	7.46 ± 0.53	8.40 ± 0.52	7.60 ± 1.02	8.00 ± 1.17	7.67 ± 2.15
5w	6.86 ± 0.98	8.55 ± 0.63	8.88 ± 2.03^b^	8.45 ± 1.58	7.62 ± 1.78
6w	7.04 ± 0.49	8.17 ± 0.60	9.16 ± 1.43^b^	8.35 ± 1.06^b^	8.25 ± 0.84^b^
7w	7.26 ± 0.42	8.43 ± 0.58^b^	9.38 ± 1.03^b^	7.68 ± 0.89	7.52 ± 0.98
8w	7.28 ± 0.48	8.62 ± 0.53^b^	7.06 ± 0.67^a^	8.63 ± 0.69^b^	9.45 ± 0.61^ab^
9w	7.00 ± 0.73	8.93 ± 0.90^b^	7.76 ± 0.77^a^	7.27 ± 0.42^a^	7.73 ± 0.38^a^
10w	6.28 ± 0.66	8.52 ± 0.62^b^	5.80 ± 0.51^a^	5.00 ± 0.66^ab^	4.98 ± 0.44^ab^
11w	6.22 ± 0.11	8.57 ± 0.84^b^	6.16 ± 0.30^a^	6.12 ± 0.18^a^	5.92 ± 0.39^a^
12w	7.40 ± 0.12	8.33 ± 0.15^b^	8.02 ± 0.15^ab^	8.08 ± 0.17^ab^	7.94 ± 0.21^ab^

## Data Availability

The data used to support the findings of this study are available from the corresponding author upon request.
